# Fast but accurate: a systematic review and meta-analysis on diagnostic performance of MRSA detection in clinical samples by using CRISPR-based rapid molecular methods

**DOI:** 10.3389/fmicb.2025.1703247

**Published:** 2025-11-18

**Authors:** Yonghui Gao, Jihong Chen

**Affiliations:** 1 Department of Healthcare-Associated Infection Control, The People’s Hospital of Baoan Shenzhen, The Second Affiliated Hospital of Shenzhen University, Shenzhen Hospital of Guangdong Provincial People’s Hospital, Hospital Infection Management Quality Control Center of Baoan District, Shenzhen, Guangdong, China; 2 Department of Nephrology, The People’s Hospital of Baoan Shenzhen, The Second Affiliated Hospital of Shenzhen University, Shenzhen Hospital of Guangdong Provincial People's Hospital, Shenzhen, Guangdong, China

**Keywords:** methicillin-resistant *Staphylococcus aureus*, CRISPR/Cas, diagnostic accuracy, point-of-care testing, rapid detection, meta-analysis

## Abstract

**Background:**

Methicillin-resistant *Staphylococcus aureus* (MRSA) poses a significant global health threat due to its multidrug resistance and association with severe infections. Conventional culture methods are time-consuming, usually requiring 48–72 h to obtain results, while conventional molecular methods such as PCR or qPCR, though faster, still require trained personnel and specialized instruments, which may delay timely clinical treatment and infection control. CRISPR-based methods have emerged as promising alternative tools for MRSA detection, but their real-world performance still requires comprehensive assessment. This meta-analysis aimed to systematically evaluate the diagnostic accuracy and timeliness of CRISPR/Cas systems for MRSA detection in clinical samples.

**Methods:**

A systematic search of PubMed, Embase, Web of Science, and Cochrane Library was conducted using search terms related to MRSA, CRISPR/Cas, diagnostic accuracy, and rapid detection. Studies reporting sensitivity and specificity with extractable 2 × 2 contingency tables were included. Quality was assessed via QUADAS-2. Meta-disc 1.4.0 and Stata 16.0 were used for statistical analysis, including pooled sensitivity, specificity, likelihood ratios, diagnostic odds ratios (DOR) and summary receiver operating characteristic (SROC). Median detection time and subgroup analyses were also conducted.

**Results:**

Twelve studies were included. The results showed that the CRISPR-based methods showed a pooled sensitivity of 99% (95% CI: 97–100%) and specificity of 100% (95% CI: 99–100%), with a PLR of 32.68 (95% CI: 15.45–69.15), NLR of 0.03 (95% CI: 0.02–0.07), and DOR of 664.25 (95% CI: 234.59–1880.84). The median detection time across included studies was 60 min (IQR: 41.25–98.75 min).

**Conclusion:**

CRISPR-based molecular assays demonstrated exceptional accuracy and rapid detection capability for MRSA in clinical settings, significantly outperforming conventional methods. However, potential publication bias and methodological limitations warrant cautious interpretation of these results.

**Systematic review registration:**

PROSPERO ID: CRD420251115439.

## Introduction

Methicillin-resistant *Staphylococcus aureus* (MRSA), a multidrug-resistant organism, has emerged as a significant global health threat due to its ability to cause severe infections and its notable resistance to multiple antibiotics, especially beta-lactams (Lee et al., 2018). MRSA infections are associated with increased morbidity and mortality, particularly in hospitals, where they can manifest as bloodstream infections, pneumonia, or surgical-site infections (Chalmers and Wylam, 2020). Actually, MRSA accounts for a substantial proportion of hospital-acquired infections and poses a challenge to effective treatment strategies and antimicrobial stewardship (Cassini et al., 2019; Fernando et al., 2017). Therefore, rapid and accurate detection of MRSA infection is essential for timely clinical treatment and infection control to limit its spread within hospital.

Conventional MRSA detection methods mainly include culture followed by susceptibility testing, and the PCR amplification of its characteristic gene, *mecA* (Rajan et al., 2007; Soderquist et al., 2012). Though these methods are considered the gold standard, they are often critically time-consuming. For example, culturing often takes 2–3 days and depends on many factors such as the quality of the clinical specimens. PCR, though faster, usually takes at least 2 h and requires specific laboratory conditions and professional knowledge of the operators (Luteijn et al., 2011). In this case, the conventional methods may delay appropriate antibiotic therapy and patient isolation, leading to further spread of the pathogen to others in the hospital. Therefore, the development of rapid, sensitive and easy-to-operate MRSA detection methods that can be implemented in timely clinical settings has become a critical focus to enhance patient care and infection control (Bakthavatchalam et al., 2017; Coia et al., 2006).

In recent years, molecular approaches have gained increasing importance in the diagnosis of various infections, addressing the urgent need for rapid diagnosis of pathogens, including MRSA (Ye et al., 2025; Palavecino, 2020; Hirvonen, 2014). These approaches use the principles of molecular biology to provide rapid, accurate, and sensitive identification of pathogens. Among them, CRISPR-based methods have emerged as powerful tools that provide a robust platform for the rapid detection of pathogens (Wang et al., 2023). Briefly, CRISPR/Cas-based detection relies on a guide RNA (gRNA) that directs the Cas protein to recognize a specific DNA or RNA sequence of the target pathogen. Once bound, the Cas enzyme becomes activated and cleaves nucleic acids through cis-cleavage of the target sequence and trans-cleavage of nearby reporter molecules. The cleavage of the reporter molecules produces detectable fluorescence or colorimetric signals, enabling rapid and highly specific identification of pathogens. Owing to the ability of CRISPR system to specifically target pathogen-associated nucleic acid sequences such as the *mecA* gene of MRSA, these methods can accurately identify the pathogens by combining with various rapid molecular amplification methods and different signal readout platforms (Peacock and Paterson, 2015).

Recent studies have demonstrated the potential of combination of CRISPR/Cas system and nucleic acid amplification techniques such as loop-mediated isothermal amplification (LAMP) and recombinase polymerase amplification (RPA) to identify MRSA in clinical samples with remarkable speed and accuracy, often within a matter of minutes (Cao et al., 2023; Liu et al., 2023). The application of CRISPR/Cas system in diagnostics not only enhances the speed of detection but also allows for the development of point-of-care testing solutions that can be deployed in diverse clinical environments, therefore significantly improving patient treatment and outcomes in MRSA infections and assist the antimicrobial stewardship.

Although many studies have revealed its promptness, sensitivity and applicability in MRSA detection, there remains a notable gap in the comprehensive evaluation of the efficiency and accuracy of CRISPR-based MRSA methods in real-world settings. Therefore, the present study aimed to perform meta-analysis and systematic review to comprehensively evaluate the diagnostic accuracy and timeliness of CRISPR/Cas-based technologies for MRSA in clinical samples.

## Methods

### Retrieval strategy

A systematic search of the PubMed, Embase, Web of Science, and Cochrane Library databases (up to May 31, 2025) was conducted using a combination of subject terms, including: (“Methicillin-Resistant *Staphylococcus aureus*” OR “MRSA”), (“CRISPR” OR “Clustered Regularly Interspaced Short Palindromic Repeats” OR “Cas”), (“diagnostic accuracy” OR “sensitivity” OR “specificity”), (“rapid detection” OR “point-of-care testing” OR “rapid diagnosis”), etc. The initial screening studies were imported and managed using NoteExpress software.

### Inclusion and exclusion criteria

Based on the PICOS principles (Amir-Behghadami and Janati, 2020), studies were included if it meets the following requirements and standards: (1) Patients (P): Clinical MRSA specimens or clinically infected MRSA patients; (2) Intervention (I): Detection methods based on CRISPR/Cas system; (3) Comparison (C): Using traditional culture methods, conventional PCR or qPCR methods as control methods as control methods; (4) Outcomes (O): Reports on sensitivity, specificity, and extractable 2 × 2 contingency table; (5) Study design (S): Diagnostic accuracy studies (prospective/retrospective cohorts) or technical validation studies (must include clinical sample validation). Reviews, conference abstracts and unpublished studies are excluded.

### Data extraction and quality assessment

The following information was extracted by two researchers independently using a pre-designed Excel spreadsheet: authors, publication year, country, study type, sample type, gold standard, CRISPR type, detection platform, number of MRSA and non-MRSA samples, true positives (TP), false positives (FP), false negatives (FN), true negatives (TN), detection limit (LOD) and detection time.

At the same time, quality assessment of each study was performed independently by the two researchers according to the principle of Quality of Diagnostic Accuracy Studies-2 (QUADAS-2), a recommended tool for evaluating studies in meta-analysis for diagnostic accuracy (Qu et al., 2018). The bias risks consist of four dimensions: Patient Selection, Index Test, Reference Standard, and Flow and Timing. The researches answered the questions of each item by “Yes,” “No” or “Unclear” and rated “High,” “Low,” or “Unclear” for bias risks.

### Statistical analysis

Meta-disc 1.4.0 and Stata 16.0 software were used for statistical analysis. The pooling sensitivity, specificity, positive/negative likelihood ratios (PLR/NLR), diagnostic odds ratio (DOR), and the summary receiver operating characteristic curve (SROC) with 95% confidence intervals were obtained by Meta-disc 1.4.0. The Fagan nomogram and Deeks’ funnel plot was produced by Stata 14.0. GraphPad Prism 8.0 was used to draw the figures.

## Result

### Literature screening process

**Figure 1:**
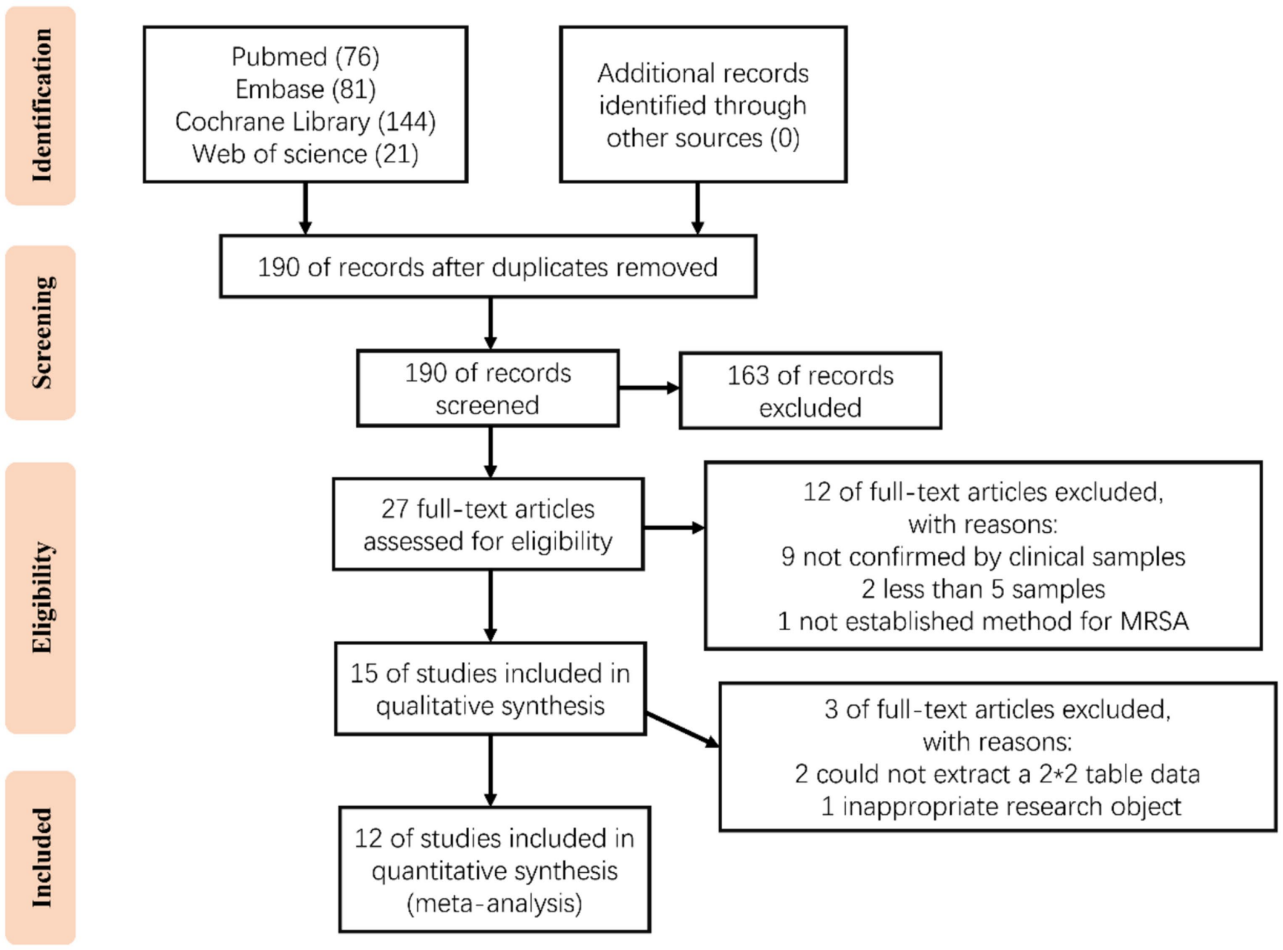
Study flow diagram.

A total of 322 articles were obtained through our systematic retrieval (PubMed: 76, Embase: 81, Web of Science: 21, Cochrane: 144). After removing the duplicates, 190 articles were retained. Based on the title/abstract content, 163 articles were excluded (including non-diagnostic studies/non-CRISPR technology/non-MRSA, etc.). Later, the remaining 27 articles were subjected to full text reading for assessing the eligibility, resulting in the exclusion of 12 articles, including 9 that clinical samples were not applied for validation; 2 with number of samples lower than 5; and 1 that did not establish a method for MRSA detection. Then, the remaining 15 studies were further subjected to qualitative synthesis, resulting in 2 studies were excluded from which the 2 × 2 table could not be extracted; and 1 was excluded because of inappropriate research objectives. Finally, 12 studies were included for meta-analysis (Figure 1) (Cao et al., 2023; Fan et al., 2025; Li et al., 2022; Sun et al., 2023; Wang et al., 2025; Wang et al., 2022; Wei et al., 2022; Wu et al., 2023; Wu et al., 2023; Zhang et al., 2024; Zhao et al., 2025; Zheng et al., 2023).

### Characteristics of included studies

A total of 13 data sets were extracted from the 12 articles and summarized, as shown in Table 1.

**Table 1:** The detailed information of the included studies.

Author, Year	Country	Study design	Sample type	Gold standard	Type of CRISPR/Cas	TP	FP	FN	TN	Detection time (min)^#^	Read out platform
Li et al. (2022)	China	Prospective	Unknown	qPCR	One-Tube RPA-CRISPR/Cas12	21	0	0	2	20	Fluorescence
Li et al. (2022)	China	Prospective	Unknown	qPCR	One-Tube RPA-CRISPR/Cas12	20	0	1	2	20	Lateral flow strips
Wu et al. (2023)	China	Prospective	Secretions	Culture and PCR	CPA-Cas12a	3	0	0	199	30	Lateral flow strips
Sun et al. (2023)	China	Prospective	Blood	qPCR	Multiplex RPA-CRISPR/Cas12a	4	0	0	8	35	Fluorescence
Fan et al. (2025)	China	Prospective	Unknown	PCR	dCas9/crRNA	4	0	0	4	60	Colorimetry
Wang et al. (2025)	China	Prospective	Multiple specimens^a^	Culture	RPA-CRISPR/Cas12a	44	0	1	45	60	Fluorescence
Wang et al. (2022)	China	Prospective	Multiple specimens^b^	Culture and PCR	RAA-CRISPR-Cas12a	41	0	0	42	60	Fluorescence
Zhao et al. (2025)	China	Prospective	Blood	Culture	CRISPR-Cas12a	27	0	0	23	60	Fluorescence
Cao et al. (2023)	China	Prospective	Unknown	PCR	mecA-LAMP-Cas12a	61	0	0	50	80	Fluorescence
Wei et al. (2022)	China	Prospective	Unknown	qPCR	RPA-CRISPR/Cas12a	4	0	0	8	95	Colorimetry
Zheng et al. (2023)	China	Prospective	Multiple specimens ^c^	PCR	RPA-CRISPR/Cas12	8	0	0	4	100	Multiplex*
Wu et al. (2023)	China	Prospective	Serum	Culture and qPCR	RPA-CRISPR/Cas12	10	0	0	10	120	Colorimetry
Zhang et al. (2024)	China	Prospective	Unknown	MIC and PCR	RCA-CRISPR/Cas12	10	0	0	5	120	Multiplex**

a Blood, urine, and bronchoalveolar lavage fluid.

b Secretions, fester, drainage fluid or indwelling catheters, sputum, blood, and urine.

c Sputum, secretin and blood.

^#^ Detection time (min) refers to the time taken by duration from the start of the detection reaction to result readout.

*Combination of fluorescence, colorimetry and photothermy.

**Combination of fluorescence, colorimetry and electrochemistry.

### Quality evaluation

**Figure 2:**
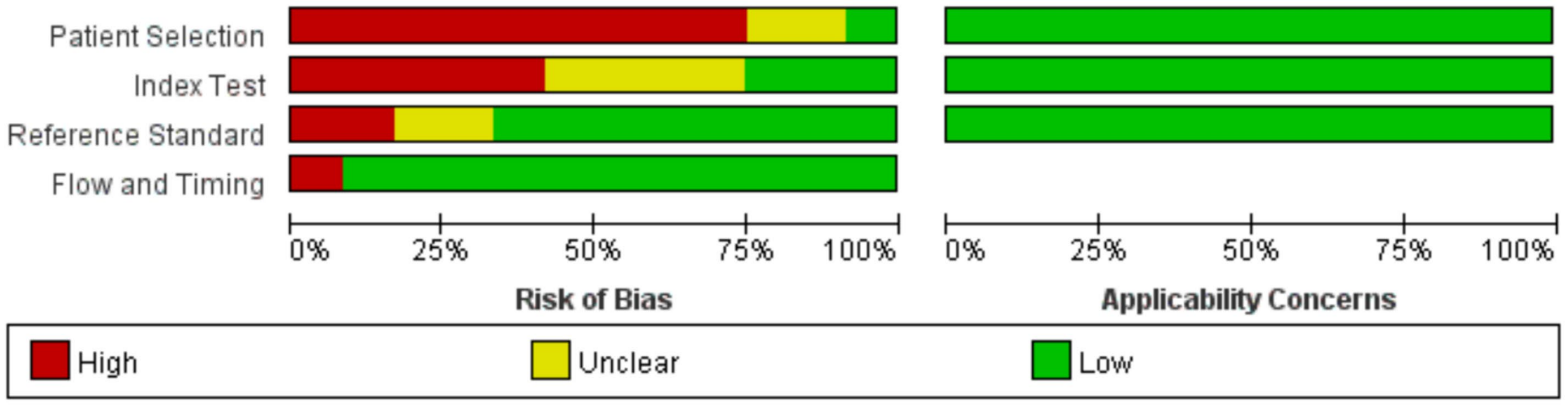
The summary of the risk of bias and applicability concerns of the included studies.

**Figure 3:**
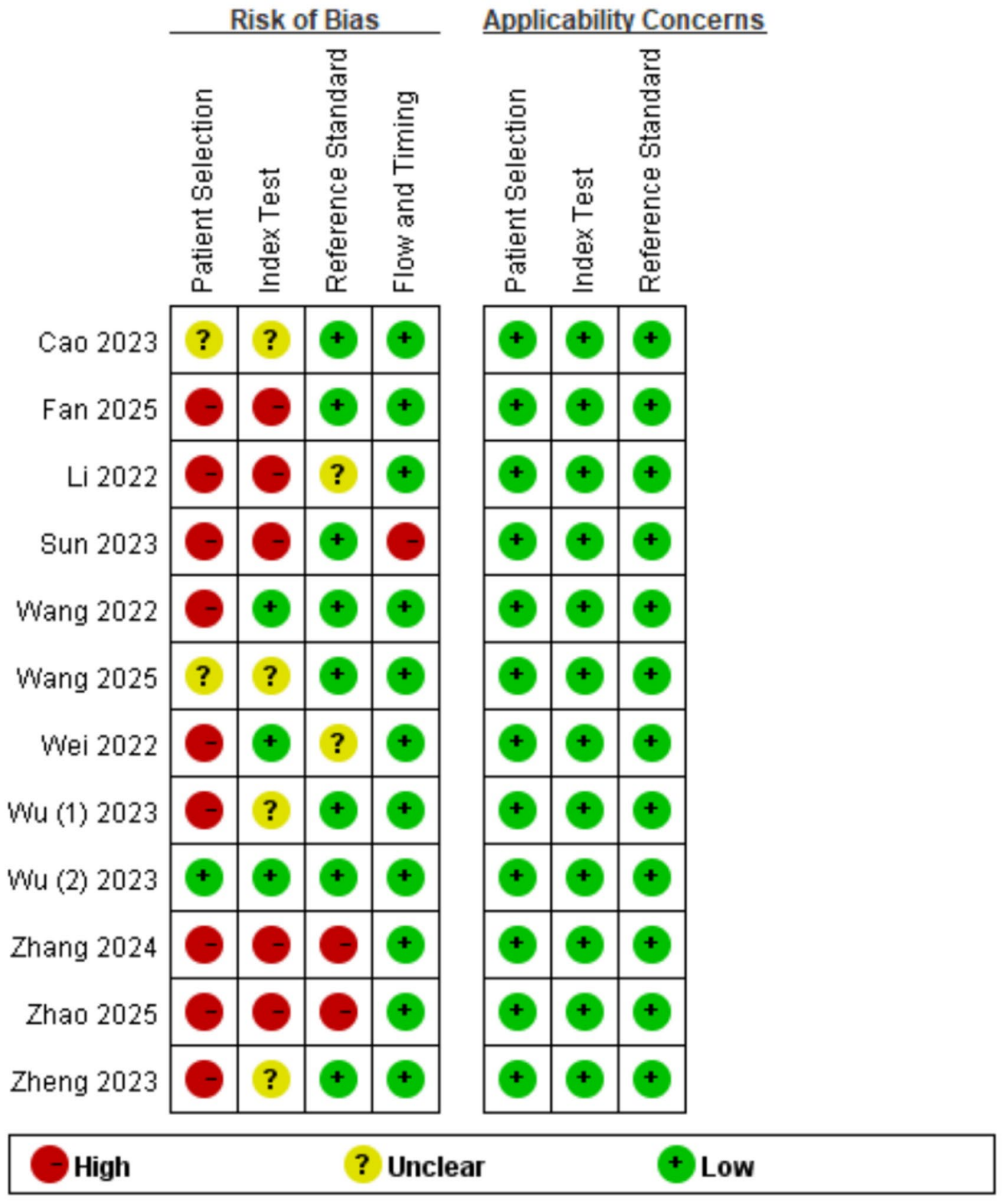
Quality evaluation of the individual studies.

Review Manager 5.4.0 was used to produce the quality plots (Figures 2, 3). In case of patient selection, 9 studies had a high risk of bias because they did not avoid a case–control design or the samples were not enrolled consecutively or randomly, and 2 studies had unclear risk; In aspect of index tests, 5 studies had high bias because the operators had already known the gold standard results, and 4 studies had unclear risk. In aspect of gold standard, 2 studies had high risk and 2 studies had unclear risk owing to their interpretation of gold standard results. One study was evaluated as high risk in terms of flow and timing due to an inappropriate interval between index test and reference standard. There was no risk in all included studies in terms of applicability concerns.

### Publication bias

**Figure 4:**
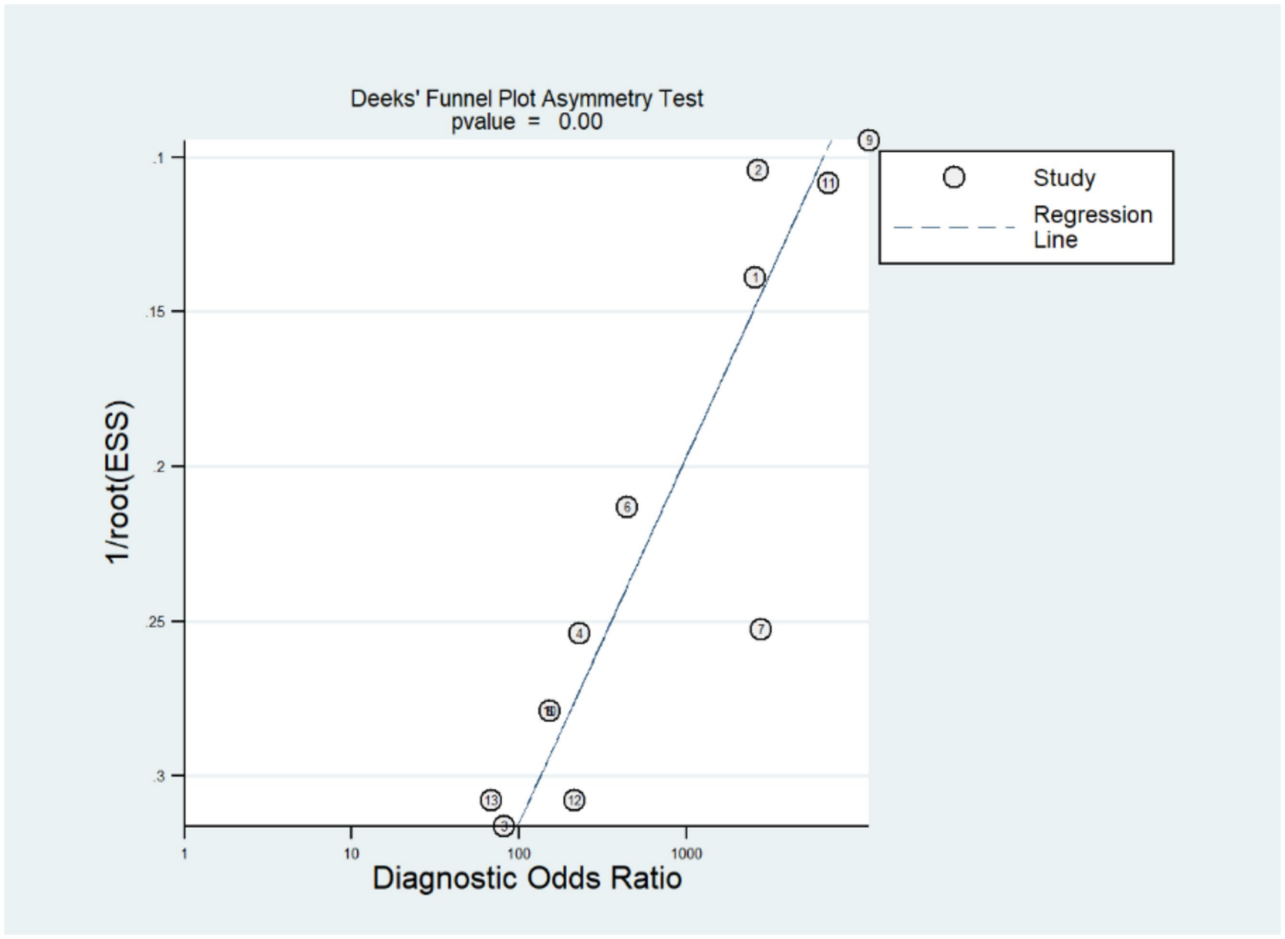
Deek’s funnel plot showing the publication bias.

The stata14.0 software was used to assess the publication bias of the included studies. According to the funnel plot (Figure 4), there was an evident publication bias in the studies we included (*p* = 0.00).

### Overall diagnostic accuracy of the CRISPR/Cas-based methods

**Figure 5:**
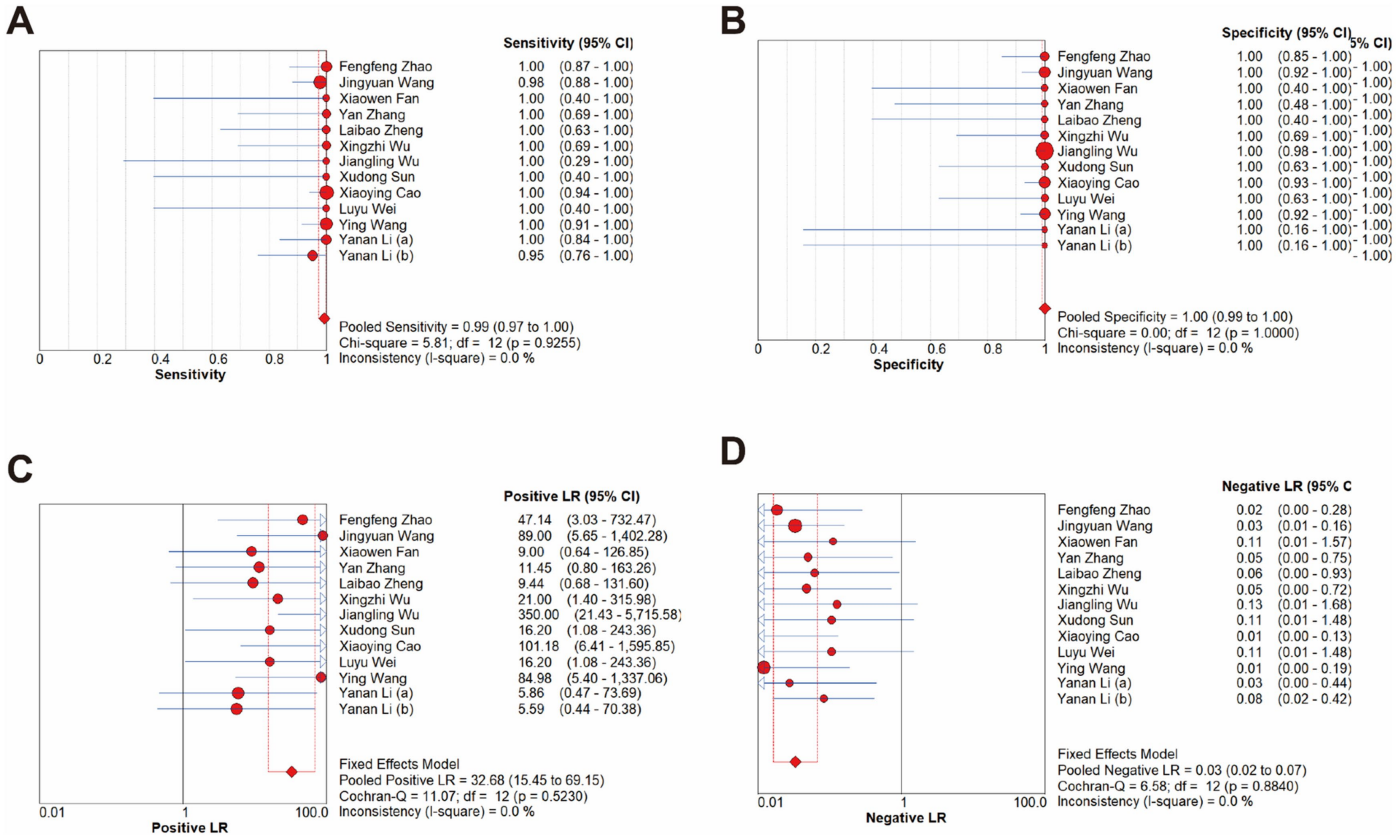
Forest plots for the pooled sensitivity **(A)**, specificity **(B)**, PLR **(C)** and NLR **(D)** of the included studies.

The overall diagnostic accuracy of CRISPR-based methods for MRSA detection in clinical samples was analyzed by meta-disc 1.4.0. The results indicated a pooled sensitivity of 99% (95% CI 97–100), and a pooled specificity of 100% (95% CI 99–100), with both the *I^2^*value of 0%. Therefore, a fixed effect model (FEM) was chosen for subsequent analysis. Results showed that the pooled PLR was 32.68 (95% CI 15.45–69.15, *I^2^* = 0.0%), the pooled NLR was 0.03 (95% CI 0.02–0.07, *I^2^* = 0.0%), and the pooled DOR was 664.25 (95% CI 234.59–1880.84, *I^2^* = 0.0%) (Figure 5).

**Figure 6:**
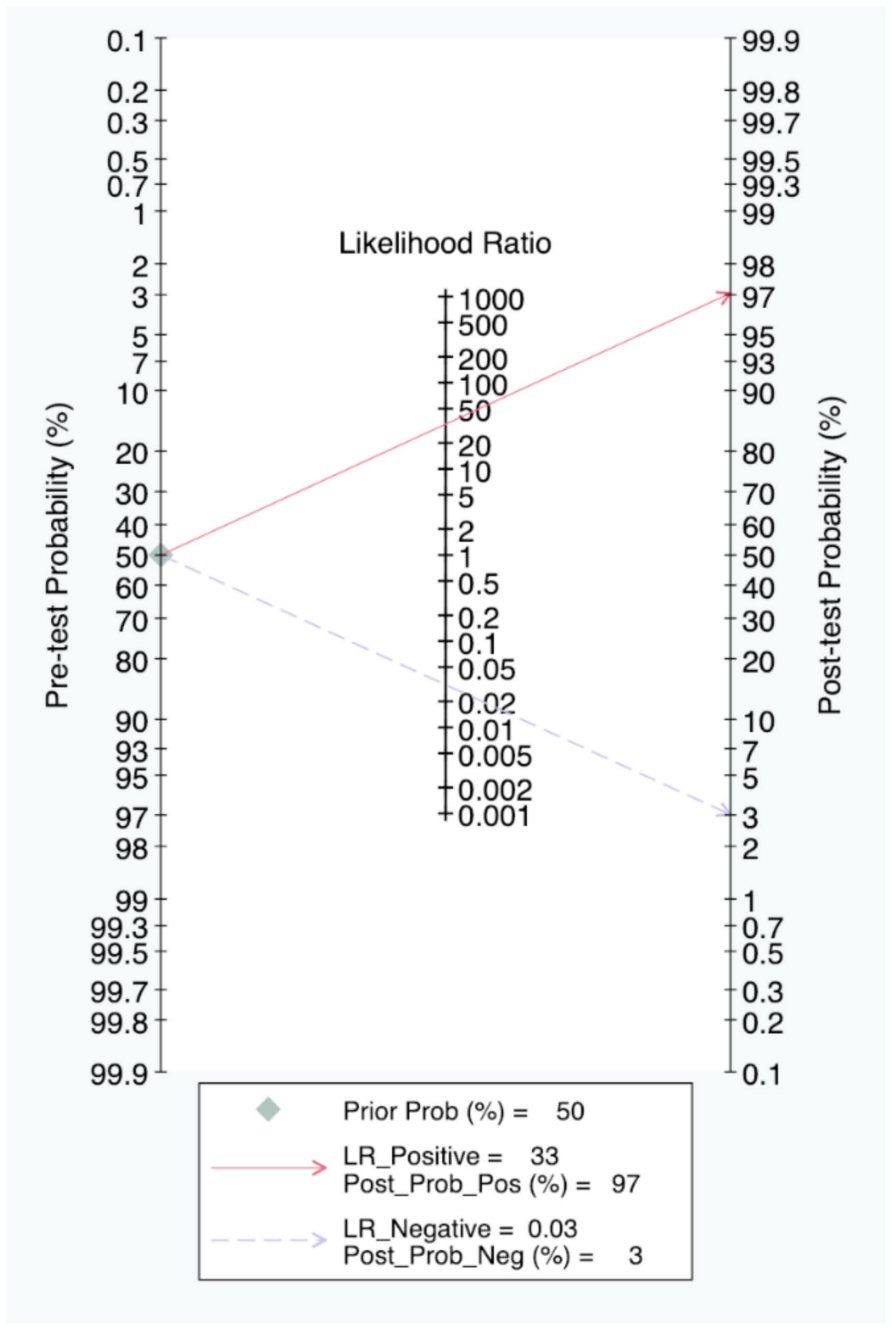
Fagan’s probability plot.

The Fagan nomogram showed that when the prior probability was 50%, the post-test probability was 97% if the results were positive, and the post-test probability was 3% if the results were negative (Figure 6).

**Figure 7:**
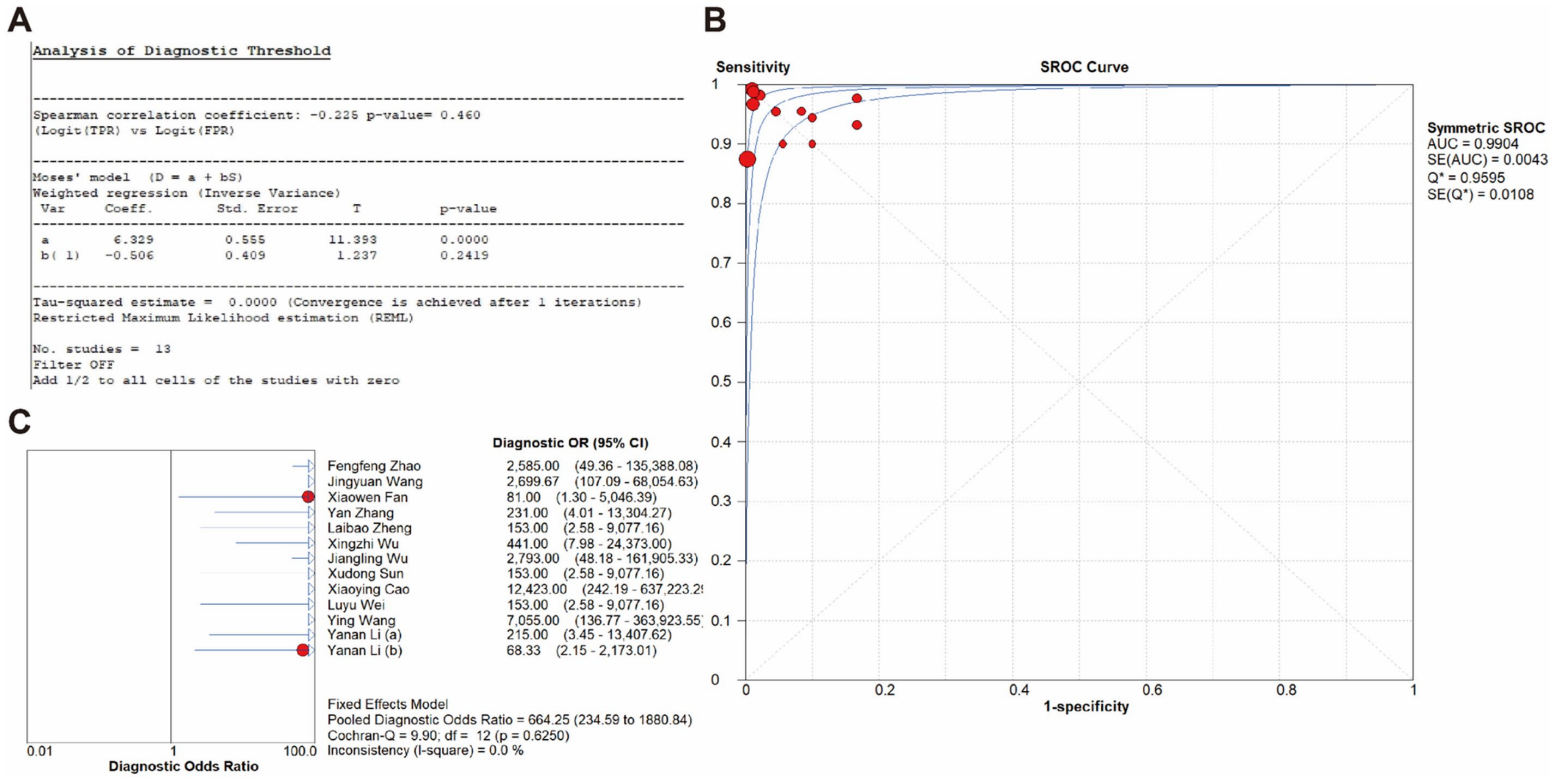
Diagnostic threshold **(A)**, SROC curve **(B)** and DOR **(C)** of the included studies.

In addition, the 2 × 2 contingency table data extracted from the included studies was imported into Meta-disc for analysis, resulting in a Spearman correlation coefficient of the log sensitivity and log (1-specificity) of −0.225, with *p*-value of 0.460, indicating that there was no threshold effect in the present study (Figure 7A). The value of *b(1)* was −0.506, with *p*-value of 0.2419, thus a symmetric SROC was selected. The area under the SROC curve (AUC) was 0.9904 and the curve did not exhibit a “shoulder-arm” shape, further confirming that there was no threshold effect in the present study (Figure 7B). What’s more, the Cochran-Q test for DOR indicated the absence of threshold effect heterogeneity, with Cochran-Q = 9.90, *p* = 0.6250, *I^2^* = 0.0%, implying that the present study did not exhibit non-threshold effect heterogeneity (Figure 7C).

### Sensitivity analysis

In order to further evaluate the robustness of the present study, a leave-one-out sensitivity analysis was conducted. The result showed that the pooled sensitivity, specificity, PLR, NLR, and DOR changed only slightly when removing any single study (Table 2), suggesting the stability of our findings.

**Table 2:** Results of leave-one-out sensitivity analysis for CRISPR/Cas-based MRSA detection.

Study omitted	Sensitivity	Specificity	PLR	NLR	DOR
None	0.992	1.000	32.685	0.033	664.250
Fengfeng Zhao	0.991	1.000	31.454	0.035	606.230
Jingyuan Wang	0.995	1.000	28.261	0.033	557.760
Xiaowen Fan	0.992	1.000	34.545	0.031	769.160
Yan Zhang	0.992	1.000	34.894	0.032	706.930
Laibao Zheng	0.992	1.000	35.086	0.032	726.730
Xingzhi Wu	0.992	1.000	33.603	0.032	680.870
Jiangling Wu	0.992	1.000	31.776	0.030	648.220
Xudong Sun	0.992	1.000	33.590	0.031	726.730
Xiaoying Cao	0.990	1.000	26.735	0.041	503.430
Luyu Wei	0.992	1.000	33.590	0.031	726.730
Ying Wang	0.991	1.000	28.629	0.038	547.530
Yanan Li	0.992	1.000	36.628	0.033	693.420
Yanan Li	0.996	1.000	36.669	0.032	797.680

### Detection time of CRISPR-based methods for MRSA

**Figure 8:**
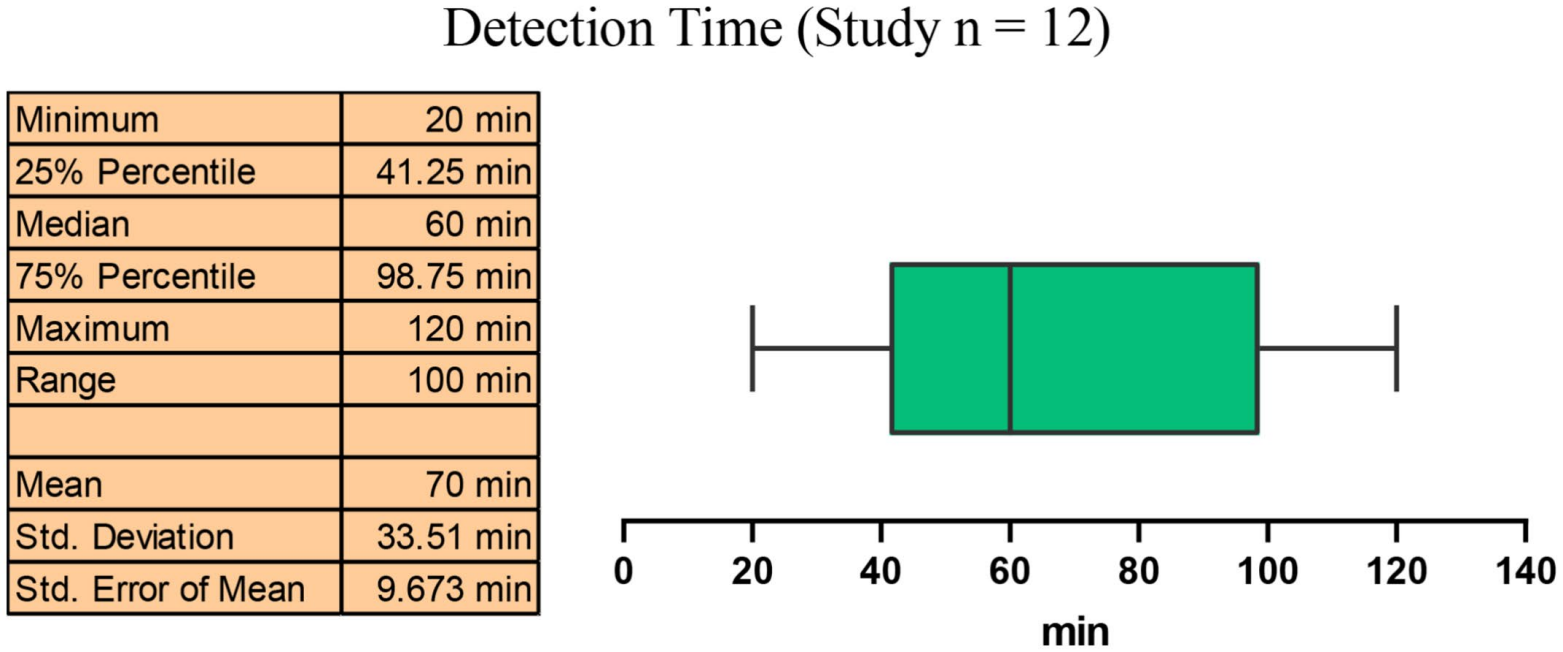
Median detection time of the CRISPR-based methods on MRSA.

Among the 12 studies we included, the median detection time for the CRISPR-based methods for MRSA detection was 60 min (IQR: 41.25–98.75 min), with the interquartile range indicating detection times within 57.5 min. Compared to traditional culture methods (which usually take 2–3 days) and traditional PCR methods (2–3 h) (Rajan et al., 2007), the CRISPR-based methods significantly shortened the detection time in less than 2 h while ensuring high sensitivity and high specificity (Figure 8).

**Figure 9:**
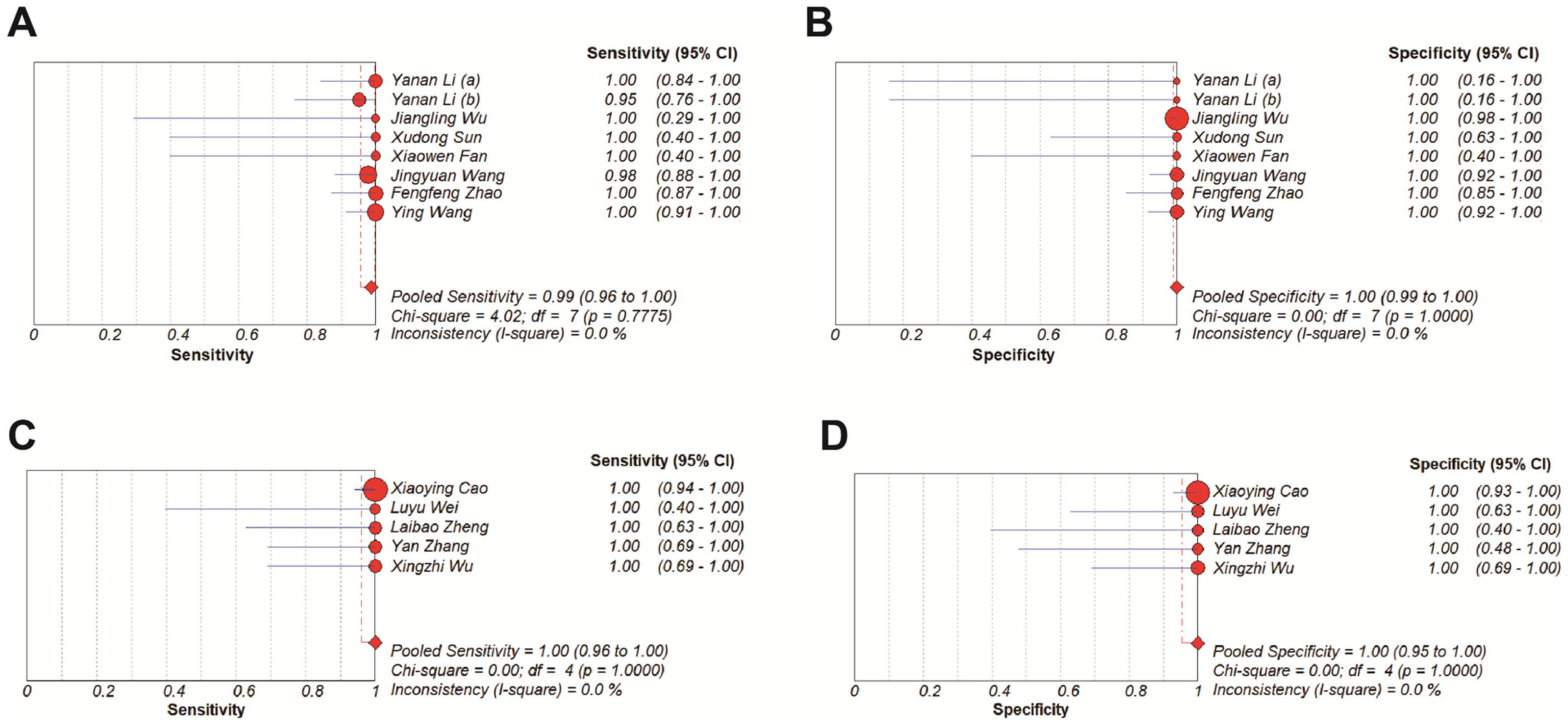
Forest plots for the pooled sensitivity and specificity of subgroup analysis. **(A,B)** ≤ 1 h. **(C,D)** >1 h.

All the included studies have a turnaround time of MRSA detection within 2 h, which largely meets the clinical demand for rapid testing (Gritte et al., 2021). In order to explore whether “speed comes at the cost of accuracy,” based on detection time, we manually separated the studies into two groups (≤1 h and >1 h) and subgroup analysis was performed. Results showed that all subgroups consistently demonstrated pooled specificity of 100%, and the pooled sensitivity was 99% in the ≤1 h group and 100% in the >1 h group (Figure 9).

## Discussion

In the present study, we performed a meta-analysis to figure out the diagnostic accuracy of CRISPR-based methods in detecting MRSA of clinical samples. Our results showed that the pooled sensitivity and specificity of the include studies was 99 and 100%, respectively. Meanwhile, the area under the SROC curve (AUC) was 0.9904, suggesting that CRISPR-based molecular methods achieved near-perfect diagnostic performance for MRSA detection in clinical samples. A similar result was got in a study analyzing the loop-mediated isothermal amplification (LAMP) assay for *Staphylococcus aureus* detection, where the AUC was 0.9976 (Long et al., 2022). Despite different detection principles, both methods are based on rapid molecular assays and achieved consistently high AUC values, indicating that CRISPR-based methods can maintain excellent accuracy under diverse conditions. Moreover, the median detection time was 60 min (IQR: 41.25–98.75 min), which is much faster than the traditional culture and PCR methods. This outstanding performance in accuracy and speed indicates that CRISPR-based molecular methods can substantially enhance diagnostic capacity and provide valuable time windows for timely clinical treatment, facilitating prompt targeted therapy to improve patient outcomes and antimicrobial management.

To be specific, in actual clinical practice, rapid and accurate detection of infections is essential for precise therapy, improving patient outcomes and assisting antimicrobial management. In our study, the pooled PLR of 32.68 and the NLR of 0.03 indicated strong diagnostic power. Furthermore, the Fagan nomogram indicated that when the pre-test probability is 50%, the post-test probability increases to 97% with a positive result, and decreases to 3% with a negative result. These results together indicated that positive results strongly increase the probability of infection, while negative results can reliably exclude it. These findings allow the clinical workers to directly see the value of CRISPR -based detection methods in the practice of clinical diagnosis, and enhance their trust in its accuracy and make appropriate clinical actions based on the results immediately.

In summary, these findings have several important clinical implications:

(1) Earlier and more targeted antimicrobial therapy: rapid and accurate detection allows clinicians to adjust antibiotic timely, shortening the time of patient to appropriate treatment. (2) Improved infection control: rapid identification of MRSA enables timely implementation of isolation or protective measures, thereby reducing the risk of hospital transmission. (3) Clinical practice: the high reliability of CRISPR-based methods increases clinicians’ confidence in early results, which may further influence perioperative preventive antibiotic strategies or therapy decisions in critical patients. (4) Economic benefits: earlier and targeted treatment can significantly reduce unnecessary empirical medication, reduce the occurrence of complications and shorten hospital stays, thereby decreasing both patient expenses and institutional healthcare costs.

Meanwhile, compared with other molecular amplification methods, CRISPR-based molecular methods have several unique advantages. Some previous studies have demonstrated the great potential of molecular amplification methods in improving the speed and accuracy of MRSA diagnosis in clinical settings (Long et al., 2022; Chen et al., 2021). In term of CRISPR/Cas systems, they are highly sensitive and specific to the target nucleic acid, and can rapidly respond after recognition. These features make it possible to combine CRISPR with various rapid amplification methods, further accelerating the detection time. Although PCR-based methods are much faster than traditional culture (2–3 days), they still require complex machines and trained operators, such as thermocyclers for 30–40 amplification cycles and agarose gel electrophoresis or fluorogenic quantitative PCR machine for result interpretation. In past few years, some efforts have been made to improve PCR-based approaches. For example, a recent study reported a multiplex TaqMan real-time PCR method, in which the thermal cycling part could be completed within approximately 30 min after pre-enrichment of blood samples (Duraiswamy et al., 2023). In addition, commercial PCR-based kits such as the Cepheid Xpert MRSA series have also been developed and widely used as point-of-care testing (POCT) for rapid MRSA detection in hospitals. Although these improved PCR methods and commercial systems have significantly shortened the amplification and detection time, some inherent limitations still exist. They rely on high-performance instruments, precise temperature control systems, and sophisticated probe designs, requiring trained personnel for proper operation. Meanwhile, the instrument cost and technical complexity remain high, limiting their POCT testing capabilities and making these systems less suitable for large-scale implementation in resource-limited or primary healthcare settings. In contrast, CRISPR-based molecular detection is much easier to operate, and it can be integrated with a variety of simple output platforms, such as fluorescence, lateral flow strips, and colorimetric assays, or even combinations of these (Kostyusheva et al., 2022). For example, among the studies we included, different platforms including fluorescence, lateral flow strips, colorimetric assays, and combinations of multiple methods were utilized (Table 1). This flexibility allows the method to adapt to different diagnostic settings, from point-of-care testing to routine laboratory use. In present study, regardless of the output platform, all CRISPR-based MRSA detection methods reported a detection time of less than 2 h, with a median of 60 min, significantly shortens the turnaround time while maintaining perfect accuracy. Of course, we also noticed a considerable variation in detection time among the included studies, ranging from 20 to 120 min. This variation could be mainly explained by the methodological differences. Firstly, as mentioned above, the output platforms differed across studies. These output platforms directly resulted in differences in total detection time due to their distinct principles, operating procedures, and reaction conditions. Moreover, different nucleic acid amplification strategies were employed, such as RPA and LAMP. These techniques are based on different reaction temperatures in principle, and the use of reagents or kits from different manufacturers may also lead to variations in reaction time and performance. Nevertheless, all CRISPR-based methods achieved a total detection time within 2 hours, fulfilling the clinical demand for rapid diagnosis. More importantly, apart from speed, the major advantages of CRISPR-based assays are their simple operation, with no need for complex laboratory instruments, and potential for POCT. Therefore, we further separated the included studies into two groups based on the median detection time, and subgroup analysis showed that even with shorter detection time (≤1 h), the diagnostic performance remains a sensitivity as high as 99%, suggesting that shortening the detection time to even within 1 hour does not compromise the accuracy of CRISPR -based MRSA detection, highlighting its potential for fast and reliable clinical diagnostics.

More importantly, early and rapid MRSA detection has important value for patient management and antimicrobial stewardship. Many studies have reported that early identification of MRSA can shorten the time to start effective therapy, reduce unnecessary use of broad-spectrum antibiotics, and assist infection control in hospitals (Mergenhagen et al., 2020; Tatli-Kis et al., 2024; Lodes et al., 2012; Parente et al., 2018). Especially in high-risk departments, such as intensive care units, rapid diagnosis can help to isolate the patient promptly and prevent the spread of MRSA to other patients. In addition, the high flexibility of CRISPR-based methods makes it possible to be developed into POCT, which is of great potential in communities or in remote areas, where laboratory equipment and trained operators are limited. At present, there are no commercial CRISPR-based detection kits specifically for MRSA. This may because most CRISPR diagnostic studies remain at the laboratory stage, and no prospective clinical trials have demonstrated their real-world performance. Moreover, some key points such as sample preparation procedures, reaction standardization and cost control remain to be addressed before clinical implementation. Similar to other newly developed molecular detection methods, CRISPR-based methods also face challenges in practical application. However, some potential improvement strategies can be taken to enhance its practical application. For example, as described in one of the studies we included, Wu et al. (2023) reported a microfluidic platform that combined the processes of isolation, amplification and detection into a microfluidic device. Therefore, further development of fully automated and portable devices could greatly promote the clinical translation of CRISPR-based diagnostics in the future. Meanwhile, more studies are also needed to explore the practical use of CRISPR-based methods in POCT settings and to provide more evidence on their effectiveness in real clinical practice. In summary, our findings highlight the promise of CRISPR-based methods for fast, reliable, and simple MRSA detection. These results support the future development of CRISPR-based POCT for MRSA, which has great potential to be further optimized for direct detection from clinical samples without complicated process. Future research should also include studies in different clinical settings to assess their real-world performance impact on antimicrobial stewardship.

At the same time, it is necessary to seriously consider several limitations that may affect the reliability and general use of the present study. First, our Deek’s test showed that a significant publication bias was present in our study. What’s more, the QUADAS-2 assessment also revealed that many studies had high or unclear risk of bias in aspect of patient selection and index test. These two problems are often related. For example, the low-quality research caused by methodological flaws such as non-randomized case selection, use of case–control designs, lack of blinding, or unclear timing and flow are easier to obtain a good-looking result. These studies are often more likely to be published, thus in turn resulting in the significant publication bias (Sterne et al., 2011). In addition, the possible false positives caused by the off-target effects of CRISPR should be noticed, which might contribute to the near-perfect sensitivity. Although none of the included studies reported this problem, it is possible that studies with such limitations remain unpublished. This could further contribute to the publication bias we observed. Together, the above two problems would both possibly result in an overestimated evaluation of the efficacy of CRISPR -based methods in MRSA detection. Therefore, we further performed a leave-one-out sensitivity analysis to evaluate the robustness of our findings. Our results showed that the pooled sensitivity ranged from 0.990 to 0.996 while the pooled specificity remained at 1.000; The PLR ranged from 26.735 to 36.669, NLR from 0.030 to 0.041, and DOR from 503.43 to 797.68. These small changes helped suggest that the overall diagnostic performance was relatively stable even after removing individual studies, indicating that our results are robust despite the presence of these biases. A similar condition was also observed in a meta-analysis that reported the diagnostic performance of LAMP assay for *S. aureus* detection (Long et al., 2022). A publication bias and the issue of article quality were both present in this study and the author also pointed out that such methodological problems may have resulted in a “beautiful” experimental result, but at the same time led to a significant publication bias when performing meta-analysis.

Overall, although our sensitivity analysis suggested the robustness of the results, the small number of included studies, methodological flaws, and potential overestimation due to publication bias remind us that the conclusions of the present study should be interpreted with caution. In addition, recent evidence has suggested a trend to discontinue routine contact isolation for MRSA in some hospital settings, as studies have indicated limited benefit for transmission prevention or patient outcomes. Nevertheless, early and accurate MRSA detection remains critical for guiding targeted antimicrobial therapy and infection control decisions (Haessler et al., 2020). Therefore, high-quality studies with more rigorous methodology and larger sample numbers are still required to provide more reliable evidence and confirm the real-world performance and clinical value of CRISPR/Cas-based MRSA detection.

## Conclusion

In summary, our study shows that CRISPR-based methods exhibit exceptional accuracy (99% sensitivity, 100% specificity) and rapid response (median: 60 min) for MRSA detection in clinical samples. Nevertheless, publication bias and study limitations warrant cautious interpretation. Future studies are necessary to validate real-world feasibility and long-term clinical impact.

## Data Availability

The original contributions presented in the study are included in the article/supplementary material, further inquiries can be directed to the corresponding author.
